# Co-developing the Child and Adolescent Mentally Healthy Classroom (CAMH Class) intervention for use in routine teaching in secondary schools

**DOI:** 10.1371/journal.pone.0359563

**Published:** 2026-10-12

**Authors:** Tracy M. Stewart, Gillean McCluskey, Hamish Johnson, Debi Fry, Lesley McAra, Josie N. Booth

**Affiliations:** 1 Moray House School of Education and Sport, University of Edinburgh, Edinburgh, United Kingdom; 2 Edinburgh Law School, University of Edinburgh, Edinburgh, United Kingdom; University of Alcala Faculty of Medicine and Health Sciences: Universidad de Alcala Facultad de Medicina y Ciencias de la Salud, SPAIN

## Abstract

Addressing the mental health needs of children and young people requires integrated and multidisciplinary action to reduce the risk of detrimental outcomes. Schools offer a unique environment for such provision. However, there is a critical need for school-based interventions that are feasible, co-designed, transdiagnostic, sustainable, and cost-effective beyond the conclusion of the trial, while aligning with pupil and teacher preferences. The current study reports the development phase of a new school-based transdiagnostic mental health intervention, embedded into routine classroom lessons, which aims to promote mental health and prevent the onset or escalation of mental health symptoms in young people. The study involved a total of 32 participants aged 15–63 years (*M*_*age*_ = 32.72, *SD* = 12.73), across three groups: one group of teachers (*M*_*ag*e_ = 36.80, *SD* = 11.29) took part in four sequential development workshops, one group of young people (*M*_*age*_ = 15.85, *SD* = 0.69) took part in one development workshop, and a new group of teachers who were not part of the initial development took part in one refinement workshop (*M*_*age*_ = 37.38, *SD* = 9.26). The study identified mental health priority areas in the classroom and brought together teachers and young people to co-develop activities that can be integrated directly into routine teaching to support and promote mental health. The intervention was deemed feasible and acceptable by both teachers and young people, subject to contextual considerations. Teachers perceived the activities to have a positive impact on young people’s mental health, engagement, learning, energy, and overall enjoyment in class. The development of this intervention, with its co-production approach, has the potential to bridge the gap in intervention translation to the classroom and ultimately benefit young people’s mental health.

## Introduction

### Mental health in children and young people

Globally, one in seven children and young people (aged 0–18) meet the criteria for a mental health disorder [1]. The World Health Organization (WHO) defines ‘adolescents’ (10–19), ‘youth’ (15–24), and ‘young people’ (10–24) [2)]. Aligning with preferred terminology [3,4], this article uses ‘children’ (<10) and ‘young people’ (10–24), unless specifying precise ages. A comprehensive global epidemiological meta-analysis revealed that over a third (34.6%) of first mental disorders occur before age 14, nearly half (48.4%) by age 18, and nearly two-thirds (62.5%) by age 25, with a peak onset occurring at 14.5 years [5]. There is also evidence across countries worldwide that life satisfaction, an indicator of subjective wellbeing (i.e., cognitive and affective evaluations of life), is in decline among 15-year-olds [6]. This decline is reflected in the UK. In Scotland, 35% of young people experience low mood or are at risk of depression, 32% of 13–15-year-olds report moderate or severe anxiety, with overall happiness at the lowest level since 1994 [7]. Similarly, data from England indicate that one in eight children and young people (aged 5–19) have a probable mental health disorder, with emotional disorders the most common [8].

Emotional disorders, whilst not having an agreed operational definition, have been characterised by frequent and intense negative emotions, including aversive reactions to emotional experience that are driven by a diminished sense of control and negative appraisal of the emotion with efforts to dampen, escape, or avoid the emotional experience, either pre-emptively or in reaction to a negative emotional state [9]. This includes, but is not limited to, anxiety and depressive disorders. Depressive and anxiety disorders share common genetic, neurobiological, and psychosocial risk factors [10–12], and as a result, these emotional disorders tend to exhibit high co-occurrence in clinical and community settings [13]. As such, there is growing recognition of the importance of transdiagnostic work given that co-occurrence is the rule rather than the exception, with approximately half of young people diagnosed with one mental health disorder meeting the criteria for at least one other [13,14]. Furthermore, longitudinal data show that childhood symptoms increase the odds of adolescent or young adult mental health disorders at least threefold [15].

Transdiagnostic theory positions emotion (dys)regulation as a key mechanism underlying the emergence and modification of diverse mental health symptoms and disorders [16]. Emotion regulation (ER) is broadly defined as the ability to influence which emotions are felt, when they occur, and how they are expressed or experienced [17,18]. The extended process model conceptualises ER as a four-stage cyclical process comprising identification (recognising the need to regulate), selection (choosing a strategy), implementation (actioning the strategy tailored to the context), and monitoring (evaluating and adjusting). These span five broad regulatory categories (situation selection, situation modification, attentional deployment, cognitive change, and response modulation), which map directly onto how a situation is encountered, attended to, appraised, or responded to, with dysregulation possible at any stage. Conversely, Gratz and Roemer define emotion dysregulation as a multidimensional construct characterised by difficulties in emotional awareness and understanding, non-acceptance of emotions, difficulties engaging in goal-directed behaviour or inhibiting impulsive behaviour when experiencing negative emotions, and an inflexible use of effective, situationally appropriate ER strategies [19]. These models collectively highlight the specific regulatory processes and strategies, and broader emotional capabilities, that can be targeted in interventions.

Accordingly, evidence indicates that reducing emotion dysregulation, irrespective of intervention type or diagnosis, reduces mental health symptoms in children and young people [20,21], while also activating developmental cascades across academic and social domains [22–24]. Transdiagnostic interventions are clinically advantageous, cost-effective, and potentially enhance feasibility and external validity by targeting shared vulnerability mechanisms across a wide spectrum of disorders and sub-clinical symptoms [25]. However, translating these interventions into practice is limited by systemic barriers within clinical care. For example, while the personal and economic impacts of mental health symptoms and disorders are widely acknowledged [26], there is ongoing pressure on mental health services to keep up with demand [27]. In Scotland, Child and Adolescent Mental Health Services (CAMHS) referrals rose by 22% between 2013 and 2018, with rejected referrals and wait times also increasing [28]. Such wait times trebled from March 2020 to March 2021 [29]. Compounding this issue, less than two-thirds of young people requiring support access professional help [8]. This is problematic, as emotional disorders such as depression and anxiety often persist into adulthood if left untreated [30].

### School-based provision

Globally, focus has been shifting beyond traditional pathways of mental health support and promotion (e.g., clinical settings) towards provision of mental health support in schools [31,32]. At policy level, there have also been calls for the development of integrated mental health provision and education within schools [33], and young people themselves have highlighted the need for mental health professionals to work with educational providers in a more effective manner [34]. Schools constitute a unique opportunity in mental health provision, particularly given existing structures and the time that young people spend in school. It has been acknowledged that integrated and multidisciplinary action is needed to reduce the risk of adverse outcomes for children and young people [35]. A recent review reported that mental health needs during this developmental stage remain largely unmet and that it would be unrealistic to consider this the responsibility of mental health professionals alone [35]. Such provision in schools can offer a continuum of integrative care, in which both mental health and educational attainment of pupils are improved [36]. While there is a wealth of school-based mental health interventions globally, not all undergo formal evaluation [37].

### School-based interventions

School-based mental health interventions can identify individual, social, and structural determinants of mental ill-health and subsequently aim to reduce risk, increase resilience, and provide supportive environments that foster and promote mental health [38]. Mental health promotion interventions aim to strengthen social and emotional skills, teach cognitive-behavioural skills, positive behaviour, social problem solving, and social inclusion, reduce mental health symptoms, among other psychological outcomes [36,39]. Such programmes not only have a positive impact on mental health, wellbeing, and social and emotional outcomes [37,40] but also on educational outcomes [41]. While approaches vary, school-based prevention interventions often involve targeting and lowering symptoms in order to reduce the risk of disorder onset [42] and can involve full, aspects of, or adapted therapeutic-based interventions such as Cognitive Behavioural Therapy (CBT), Mindfulness Interventions (MI), Interpersonal Psychotherapy (IPT), Acceptance and Commitment Therapy (ACT), Dialectical Behaviour Therapy (DBT), and psychoeducational approaches as well as many others [42,43]. While distinctions have been made between promotion, prevention, and treatment interventions in schools, these boundaries can be challenging to draw. For example, individuals experiencing symptoms or disorders can still achieve a sense of wellbeing or reduced symptoms, which suggests that promotion and preventative interventions can be relevant across the stages of asymptomatic, at risk, distress or subclinical symptoms, to acute mental health disorders [44,45].

Despite growing interest in school-based mental health interventions, their widespread implementation encounters systemic and operational barriers. For instance, a recent review of universal school-based interventions in the UK found neutral-to-small effects in promoting emotional or mental wellbeing, with fewer positive outcome effects within secondary school populations [39]. Although universal interventions typically yield smaller effects than targeted approaches [43], they can be less resource-intensive because they bypass the need for clinical screening and identification [46]. Furthermore, universal models can bolster factors associated with positive mental health, wellbeing, and help-seeking [47]. While evidence supports their preventative capacity for depression and anxiety, using symptom reduction as a proxy for presentation, these effects frequently dissipate over time [42]. Indeed, other reviews conclude that when accounting for risk of bias and small effects, evidence for the prevention of depression and anxiety remains insufficient [43]. Crucially, however, while these interventions face criticism for small statistical effects, such outcomes often translate into meaningful improvements at the population level [48,49].

A fundamental, yet common pitfall in intervention design is the incompatibility and unsustainability beyond the conclusion of traditional intervention trials [50,51]. Implementation is frequently hindered by logistical and time constraints, challenges regarding programme fidelity and effectiveness, and stigma associated with partaking in mental health related school-based interventions [39,52,53]. A review of UK school-based universal intervention literature concluded that more robust, rigorous, and long-term research is required, which would require assessments of fidelity, validated measures of change, tracking of adverse effects, and evaluation of both qualitative and quantitative data [39]. Consequently, the research community has emphasised the necessity of integrating mental health promotion interventions directly into routine, natural classroom environments [36], while urging closer consideration of the broader structural context to better understand how school-based interventions can be successfully embedded and sustained [53].

### Co-production approach

Crucially, the active involvement of young people in the design of these interventions is imperative, facilitating a paradigm shift from passive recipients to active agents in decision-making. Indeed, involving children and young people in decisions regarding their mental health was identified as a top 10 priority area in a formal priority-setting partnership exercise [34]. Active engagement is also a basic human right, dictating that children and young people should participate in the design of policies that aim to serve them [54]. This model of shared decision-making integrates empirical evidence with participant preferences, and research indicates that this enhances self-efficacy and self-esteem, engagement with treatment, better outcomes and greater satisfaction [55,56]. Furthermore, past reviews of school-based interventions suggest that the most effective include increased teaching of mental health skills, a focus on positive mental health, and linking with academic learning in a multi-modal way [37]. Extending this co-design approach to teachers addresses historical barriers concerning low levels of acceptability in teacher-led interventions [57], and aligns with recent research emphasising that mental health interventions must evaluate their acceptability to teachers [58]. Ultimately, collaborative co-production enhances scientific rigour, ensures greater applicability and acceptability to policy and practice, and facilitates mutual learning [59]. There is an acute need for school-based interventions that are feasible, co-designed, transdiagnostic, sustainable, and cost-effective, and that align with young people and teacher preferences and values.

### The CAMH Class intervention

Following the Medical Research Council (MRC) framework for complex interventions [60] and guidelines from the INDEX study on intervention development, this study [61] reports the development phase of a novel, school-based transdiagnostic mental health intervention designed for integration into routine teaching. The intervention aims to promote mental health and prevent the onset or escalation of mental health symptoms in young people. Grounded in transdiagnostic theory, which shows that diverse mental health symptoms stem from shared core psychological processes [62–64], the CAMH Class intervention targets ER as its primary mechanism of change. It advances the field by operationalising this transdiagnostic mechanism at the environmental level through routine-embedded school lessons across the school day and within differing school subjects, providing a robust and unique theoretical rationale. The high malleability of ER, combined with teachers’ inherent role as co-regulators [65], positions the classroom environment as ideal to learn and practise ER and embed these skills into daily life.

By targeting core malleable transdiagnostic processes underlying many mental health symptoms and disorders, embedded into routine teaching, this approach may optimise the breadth of symptoms prevented. Furthermore, targeting overlapping risk factors simultaneously improves cost-effectiveness, scalability, and long-term sustainability within school ecosystems. Such systemic integration would be of particular interest to schools given the acute lack of time in the curriculum available to teachers to implement isolated mental health initiatives [66,67]. This approach further addresses recent calls in education for research-based frameworks to support how teachers manage emotional dynamics [68].

To date, no school-based intervention embedded directly into routine lessons targets ER as a transdiagnostic factor to promote mental health and prevent the onset or escalation of mental health symptoms. Centering the intervention on ER allows for backward mapping and collaboration to optimise ER capabilities and strategies within routine classroom lessons, recognising that complex interventions are most effective when adapted to context and participant needs. While the intervention targets individual skills and abilities, it adopts a socio-ecological lens by acknowledging that the mechanisms operate within and are sustained by the broader school environment. A preliminary programme theory was developed (see Table 1) to outline these mechanisms, mapping how teacher behaviour change (how: altering teaching practice) within daily classroom contexts (where: embedded directly into lessons) activates ER as a transdiagnostic mechanism for change (why: improving ER will improve mental health).

**Table 1:** A preliminary programme theory.

If (activities)
· Teachers collaboratively share priority areas, challenges, and opportunities to design and develop the programme. · Teachers embed emotion regulation activities into everyday routine classroom lessons (e.g., psychoeducation, reframing, perspective taking, problem solving, adaptive self-talk, coping statements, movement-based regulation, physiological de-escalation, and attentional deployment practices such as grounding, mindfulness, muscle relaxation, and strengths-based reflections).
**Then (mechanisms)**
· Shift in shared understanding and teacher practice occurs. · Activation of core change mechanisms: ER strategies (heightened acquisition of skills in flexible, goal-directed selection and use of strategies) and ER capabilities (heightened ability to understand, experience, and respond to emotions).
**So that (proximal outcomes)**
· Improved teacher mental health literacy and self-efficacy. · Improved pupil ER repertoire, effective and flexible use of strategies, and a decrease in emotion dysregulation.
**So that (distal outcomes)**
· Increased mental wellbeing and quality of life in pupils. · Reduced frequency, intensity, and/or onset of anxiety, depression symptoms and/or general internalising symptoms in pupils.

Given that intervention development is a dynamic, iterative process of co-design and refinement, which incorporates ideas on content, format and delivery while gathering perceptions on challenges, solutions, acceptability, feasibility, scalability, and potential impacts [61], our research questions are embedded within three stages of development: 1) co-development with teachers and young people; 2) perceptions of feasibility, acceptability, scalability, and impact with teachers; and 3) programme refinement with a new group of teachers not involved in the initial development.

**Stage 1: Co-development (teachers and young people)**

1. What are teachers’ mental health priority areas, challenges, and opportunities to promote and support mental health in the classroom?
2. What activities to support and promote mental health could be developed and adapted into routine teaching activities?
3. What are teachers’ and young people’s perceptions of the feasibility and acceptability of the teaching activities?

**Stage 2: Perceptions of feasibility, acceptability, scalability and impact (teachers)**

1. What perceived impact do the activities have in the classroom?
2. How do the activities contribute to change in the practice of teachers?
3. How scalable do teachers think the activities are to wider teaching?
4. What impact did taking part in the workshops have on teacher mental wellbeing, perceived stress, and on their mental health literacy?

**Stage 3: Programme refinement (teachers)**

1. Following amendments from stage 2, what are the perceptions of teachers’ who have not been involved in the initial workshops, on the feasibility, acceptability, scalability, and potential for practice change of the teaching activities?

## Method

### Study co-design

The current study applied a collaborative partnership and co-production approach. The research team partnered with the Scottish Youth Parliament (SYP), a youth-led, democratic organisation representing the young people of Scotland, which was responsible for facilitating the recruitment and management of young people to the project. An advisory panel was set up, comprising (a) three secondary school teachers and (b) three Members of the Scottish Youth Parliament (MSYPs). Each member of the advisory panel received a gift voucher in recognition of their time. While not a formal member of the advisory panel, a consultant clinical psychologist also provided guidance on potential intervention components.

### Participants

A total of 32 participants aged 15–63 years (*M*_age_ = 32.72, *SD* = 12.73), across three groups, took part (see Table 2).

**Table 2:** Demographic and schoollevel information.

	4 x 2-hour initial teacher group workshops (*n* = 10)	1 x 2-hour young person group workshop (*n* = 7)	1 x 2-hour new teacher group workshop (*n* = 15)
**Gender, *n* (%)**			
Male (%)	3 (30%)	1 (14.29%)	2 (13.33%)
Female (%)	7 (70%)	6 (85.71%)	13 (86.67%)
**Age,** M, SD, range	M_age_ = 36.8, SD = 11.29 Range = 28–63	M_age_ = 15.85, SD = 0.69 Range = 15–17	M_age_ = 37.38, SD = 9.26 Range = 25–57
**Ethnicity, *n* (%)**			
White	10 (100%)	5 (71.43%)	14 (93.33%)
Asian, Scottish Asian, or British Asian	0 (0%)	1(14.29%)	0 (0%)
African, Scottish African, or British African	0 (0%)	0 (0%)	0 (0%)
Other ethnic group	0 (0%)	1 (14.29%)	1 (6.67%)
**The Scottish Index of Multiple Deprivation (SIMD) based on home postcode data, *n* (%)**			
1 (most deprived)	0 (0%)	1 (14.29%)	0 (0%)
2	2 (20%)	3 (42.86%)	0 (0%)
3	3 (30%)	0 (0%)	3 (20%)
4	3 (30%)	2 (28.57%)	4 (26.67%)
5 (least deprived)	2 (20%)	1 (14.29%)	8 (53.33%)
**Lived experience of receiving mental health support, *n* (%)**			
Yes (%)	5 (50%)	5 (71.43%)	9 (60%)
No (%)	5 (50%)	2 (28.57%)	6 (40%)
**Subjects taught**	Geography, Environmental Science, Business Education, Personal and Social Education, Health and Wellbeing, Leadership, English, Chemistry, Biology, Gaelic, History, Childcare, Art, Design, Mathematics, Drama, Religious Moral Education, Philosophical Studies, Psychology, Design Technology and Graphics	N/A	Physics, Maths, English, Biology, Science, Health and Wellbeing, Religious, Moral and Philosophical Studies, Outdoor Education, History, Early Education and Childcare, Chemistry, and Geography
**Number of local authority areas**	9	6	8
**Years since qualified,** M, SD, range	M_years_ = 11.2, SD = 9.11 Range = 1–30	N/A	M_years_ = 12.83, SD = 2.16 Range = < 1–25
**Years taught**, Range	Range = 1–6	N/A	Range = 1–6
**Mental health related role in school** (e.g., mental health first aider, pupil support), ***n* (%)**			
Yes (%)	5 (50%)	N/A	6 (40%)
No (%)	5 (50%)	N/A	9 (60%)
**School year, *n* (%)**			
S4	N/A	1 (14.29%)	N/A
S5	N/A	4 (57.14%)	N/A
S6	N/A	2 (28.57%)	N/A

SIMD: The Scottish Index of Multiple Deprivation (SIMD is a relative measure of deprivation derived from home postcodes, ranging from quintile 1 (the 20% most deprived data zones in Scotland) to quintile 5 (the 20% least deprived data zones in Scotland).

### Measures

Measures were dependent on the stage of the project. See Table 3 for measures at each stage of the project.

**Table 3:** Measures for each stage of the project by group.

Stage 1: Co-development	Group(s)	Measure
Mental health lived experience (pre-workshop Qualtrics)	Teachers (both groups) Young people	Assessed by a single question edited from the OxWell study [69]: “Have you ever received any mental health support? For example, from CAMHS/ private psychologist/ counsellor/ charity service/ GP.” The response options were: 0 = no; 1 = yes.
Workshops	Teachers (both groups) Young people	The workshops consisted of interactive large and small group activities and discussions, breakout rooms, interactive polls and games, and a semi-structured focus group interview (see supplemental material for interview questions).
**Stage 2: Perceptions of feasibility, acceptability, scalability, and impact**	**Group(s)**	**Measure**
Workshops	Teachers (both groups)	The workshops consisted of interactive large and small group activities and discussions, breakout rooms, interactive polls and games, and a semi-structured focus group interview (see S1 Table for interview questions).
Mental Health Literacy (pre-post workshops Qualtrics)	Teachers (initial development group only)	Assessed using the educator version [70] of the mental health literacy scale: MHLS [71]. The MHLS consists of 35-items and measures participants’ knowledge of areas of mental health, and attitudes towards mental health and help seeking. The educator version of the MHLS has 5 alternative items which better fit educational situations and consists of both dichotomous and Likert items resulting in total score from 35 to 160, with higher scores indicating higher levels of MHL. The MHLS education version demonstrated good internal consistency of Cronbach’s α = . 74 at T1 and Cronbach’s α = .84, and a good test-retest reliability of *r* = .74.
Perceived Stress (pre-post workshops Qualtrics)	Teachers (initial development group only)	Assessed using the 10-item Perceived Stress Scale: PSS [72]. The PSS-10 assesses how unpredictable, uncontrollable, and overloaded one perceives their life to have been in the previous one month. Each item has 5 response categories: ‘0 = never’; ‘1 = almost never’; ‘2 = sometimes’; ‘3 = fairly often’; and ‘4 = very often’, resulting in total scores from 0 to 40 with higher scores indicating higher perceived stress. The PSS-10 demonstrated an excellent internal consistency of Cronbach’s α = .90 at T1 and Cronbach’s α = .89 at T2, and a moderate test-retest reliability of *r* = .54.
Wellbeing (pre-post workshops Qualtrics)	Teachers (initial development group only)	Assessed using the Short Warwick-Edinburgh Mental Well-being Scale: SWEMWBS [73]. The 7-items assess the participants thoughts and feelings over the previous two weeks, with more of a focus on functional thoughts and feelings compared to the 12-item version. Each item has 5 response categories: ‘none of the time’; ‘rarely’; ‘some of the time’; ‘often’; and ‘all of the time’, resulting in a total score from 7 to 35, with higher total scores indicating a higher positive mental wellbeing. The SWEMWBS demonstrated excellent internal consistency of Cronbach’s α = .91 at T1 and Cronbach’s α = .92, and a good test-retest reliability of *r* = .75.
Perceived practice change, scalability, usability, and impact (post workshop Qualtrics)	Teachers (initial development group only)	Assessed by Qualtrics open textbox survey questions: a) “Please tell us how usable you feel the strategies developed for the mental health resource are for your classroom(s)?”; b) “Please tell us about any changes to your practice following on from the strategies developed?”; c) “Please tell us about any impact for pupils you have noticed following this practice change(s)?”; and d) “Please tell us how scalable you think the strategies are (e.g., use in different subjects, year groups etc)?”
**Stage 3: Programme refinement**	**Group(s)**	**Measure(s)**
Workshop		The workshops consisted of interactive large and small group activities and discussions, interactive polls and games, flip charts, post-it-notes.
Perceived practice change, scalability, and usability (post workshop Qualtrics)	Teachers (new programme refinement group only)	Assessed by Qualtrics open textbox survey questions: a) “Please tell us how usable you feel the strategies discussed in the workshop are for the classroom(s)”; b) “Please tell us how scalable you think the strategies discussed in the workshop are (e.g., use in different subjects, year groups etc.)”; and c) “Please tell us about any ideas you have to change your practice following on from the workshop.”

### Procedure

Ethical approval was obtained from the Moray House School of Education and Sport Research Ethics Committee. A total of 6 x 2-hour workshops were conducted, across three groups, adhering to data saturation recommendations [74,75]. The advisory panel advised on the workshops design and measures, and interview questions were piloted with the group. The recruitment period was between 5^th^ September 2022 and 23^rd^ June 2023. Written informed consent from teachers and consent/assent for MSYPs was collected via Qualtrics at the start of the study and continued oral informed consent/assent gained at each workshop. The SYP coordinated all MSYP recruitment and involvement in accordance with their established youth research policies. These procedures included providing parents/guardians with study information, an opt-out consent option, and contact details for both the research team and an independent complaints liaison. A total of four workshops were held with one group of teachers, one workshop with a group of young people, and one further programme refinement workshop with a new group of teachers not involved in the initial development. The participants were not known to the interviewers. Prior to participation, attendees were informed that they would not be asked about their own mental health, and no sensitive disclosures occurred. Nevertheless, a robust safety protocol for managing sensitive disclosures was established and pre-study information on data protection and limits of confidentiality was shared with participants. A SYP staff member was present during the MSYP workshop in accordance with SYP safeguarding policies. In the event of a disclosure (MSYP or teacher) the SYP/designated second project researcher was prepared to follow-up, escalate, and pass information to the relevant authorities (e.g., parents/carers, the police, or the ambulance service). To maintain a supportive and safe workshop space, all participants received pre-study support services signposting, and reminders about consent and withdrawal procedures were provided pre-and-post workshops. Workshops commenced with co-constructed ground rules and optional wellbeing activities were incorporated.

The order and aim of each workshop were as follows:

Workshop 1 (teacher group): To capture teachers’ perspectives on mental health priority areas in the classroom and on perceived challenges and opportunities to support and promote mental health in the classroom.

Workshop 2 (teacher group): To co-create a list of feasible and acceptable classroom activities based on the findings from workshop 1, the research literature, and with consultation with the advisory group.

Workshop 3 (young people group): To share the activities developed, capture views on what, if any, activities would be feasible and acceptable to young people in a classroom setting, and to co-develop adaptations to, or new additions to, the activities.

Workshop 4 (teacher group): To share adapted and new activities and feedback from the young person workshop, to ensure feasibility and acceptability for teachers. Following final agreement, teachers were asked to integrate the activities into their lessons, trial them over a four-week period, and report back on the application, perceived feasibility, scalability, and impact of embedding strategies in routine teaching activities.

Workshop 5 (teacher group): To gather user validation and feedback on the perceived application, feasibility, scalability, and impact of embedding strategies in routine teaching activities.

Workshop 6 (new teacher programme refinement group): To engage a new group of teachers, who had not yet been involved in the initial development workshops, to receive feedback on the perceived application, feasibility, acceptability, scalability, and impact of embedding strategies in routine teaching activities and to gather any new co-developed adaptations, or new additions to, the activities.

Initial broad strategies, forming the intervention components, were selected based on participants’ responses in workshop 1 and in combination with related research literature and theory: a) identifying unhelpful thinking patterns, formulating more realistic counter-thoughts, putting thoughts into perspective, not thinking the worst, fact checking, alternative interpretations and evidence gathering, and balancing positive and negative based on aspects of CBT, mindfulness and acceptance and mindfulness therapy [76–78]; b) learning adaptive problem-solving attitudes and skills based on aspects of problem solving therapy [79]; c) learning to change responses if an urge does not fit the facts or when usual action is inappropriate based on aspects of DBT [80,81]; d) cultivating gratitude and identifying and reflecting on strengths based on aspects of positive psychology and DBT [80–82]; e) focusing attention back to the present moment using mindfulness-based relaxation techniques and/or muscle relaxation based on aspects of mindfulness and DBT [80,81]; f) establishing rapport through open questions, affirmation, reflective listening, and summary reflections based on aspects of motivational interviewing [83]; and g) physically active learning to integrate movement within delivery of classroom academic lessons [84] and short physically active breaks aimed at increasing physical activity [85]. While therapeutic intervention approaches differ, they typically converge on a common mechanism to strengthen adaptive emotion, and thus, making them relevant for the current intervention. These were then co-developed into activities, accompanied with emotion related psychoeducation, suitable for routine lessons and refined and added to throughout workshops. Participants received a gift voucher in recognition of their time. Teachers also received a Continuing Professional Development (CPD) certificate for their participation. See Table 4 for full procedures at each stage of the project.

**Table 4:** Procedure for each stage of the project.

Stage 1:	Group(s)	Procedure
Co-development	Teachers (*n* = 10)	• Recruitment: facilitated by Moray House School of Education and Sport Professional Learning Office (PLO) via social media, newsletters, and a mailing list of qualified teachers across Scotland (*n* = 1800+). Interested secondary school teachers completed an expression of interest form through a dedicated project website. The purposive selection of teachers was based on gaining a sample of broad subject areas, school council areas, and years in service. Selected participants were then invited to participate via email. All invited participated and none dropped out. • Pre-workshop questionnaire completed via Qualtrics obtaining informed consent, demographic information and lived experience. • Pre-workshop questionnaire completed via Qualtrics on measures of mental wellbeing, mental health literacy and perceived stress. 3 x 2-hour virtual (audio and video) workshops held on Microsoft Teams and led by HJ, with TMS also present. The workshops activities (see Table 3) were recorded and later transcribed by HJ (and accuracy checked by TMS) for analysis.
	Young people (*n* = 7)	• Recruitment: determined, recruited and coordinated by SYP who have extensive expertise in research and working with young people and their families, and in line with their policies and procedures for their young people taking part in research. • Pre-workshop questionnaire completed via Qualtrics obtaining informed consent, demographic information and lived experience. • 1 x 2-hour virtual (audio and video) workshop with young people were held on Microsoft Teams and led by HJ, with TMS also present. The workshops activities (see Table 3) were recorded and later transcribed by HJ (and accuracy checked by TMS) for analysis.
**Stage 2:**	**Group(s)**	**Procedure**
Perceptions of feasibility, acceptability, scalability, and impact	Teachers (*n* = 10)	• Recruitment: as per Stage 1. • 1 x 2-hour virtual (audio and video) workshops (stage 1 group) were held on Microsoft Teams and led by HJ, with TMS also present. The workshops activities (see Table 3) were recorded and later transcribed by HJ (and accuracy checked by TMS) for analysis. • Post-workshop questionnaire completed via Qualtrics on measures of mental wellbeing, mental health literacy and perceived stress. • Post-workshop questionnaire completed via Qualtrics on measures of practice change, scalability, usability and perceived impact.
**Stage 3:**	**Group(s)**	**Procedure**
Programme refinement	New group of teachers (*n* = 15)	• Recruitment: as per Stage 1. • Pre-workshop questionnaire completed via Qualtrics obtaining informed consent, demographic information and lived experience. • 1 x 2-hour in-person workshop held at a University in Scotland and led by HJ, with TMS also present. The workshops activities (see Table 3) were recorded digitally (e.g., polls) and non-digitally (e.g., on flip-charts, post-it notes) and later transcribed by HJ (and accuracy checked by TMS) for analysis. • Post-workshop questionnaire completed via Qualtrics on measures of practice change, scalability, usability and perceived impact.

### Analytical strategy

The qualitative analysis followed the phases of reflexive thematic analysis as outlined by Braun and Clarke [86,87] and followed the COREQ: Consolidated Criteria for Reporting Qualitative Research checklist [88]. Given the exploratory nature of this project, inductive coding was used. Two authors (TMS, PhD, lecturer, female; HJ, PhD, postdoctoral researcher, male) familiarised themselves with the data and worked collaboratively to generate initial codes. Collated codes were subsequently abstracted into potential themes. In the last phase, a coding tree was created (S2 Table) and five themes were established. In line with Braun and Clarke’s (2021) recommendations that researchers make clear their personal positionality the coding was shaped by the team’s positionality, with TMS and HJ drawing on their backgrounds in psychology and mental health, actively reflecting on how their assumptions influenced analytical decisions.

To maintain methodological rigour and avoid forcing the data into a purely psychological framework, codes and themes were critically discussed, reviewed, and refined in dialogue with a third author (GM, PhD, professor, female) who contributed a positionality grounded in educational leadership and inclusion. TMS and HJ are experienced and trained in quantitative and qualitative methods and analysis and GM is experienced and trained in qualitative methods and analysis. All subsequent authors (PhD, senior lecturers and professors, female) had an opportunity to review the themes and provide interpretations of the data, ensuring that multiple disciplinary viewpoints informed interpretation, supporting transparency and credibility. Trustworthiness and rigour were established through reflexivity, all author engagement, investigator triangulation, and peer debriefing, which supported analytical decision-making. This allowed for multidisciplinary triangulation to interpret the data, to bring dialogue from different academic and professional backgrounds and perspectives, challenge any psychological assumptions and offer alternative, nuanced interpretations of the data. Participants did not check transcripts or provide feedback on the findings. The sample was considered adequate for the intervention development aims and the final workshop deliberately examined the refined intervention with an independent teacher group.

NVivo20 was used to organise and facilitate the analysis. Paired-samples *t*-tests were conducted in SPSS (version 29) to examine potential pre-and-post workshop differences in teachers’ mental health literacy, perceived stress, and a Wilcoxon signed-rank test to examine potential mental wellbeing differences. The effect sizes were calculated using the sample standard deviation of difference scores for the paired-sample *t*-test and the rank-biserial correlation for the Wilcoxon signed-rank test. No separate quantitative power calculation was undertaken, and the quantitative sample size was determined by the size of the intervention-development teacher group rather than by power to detect pre-post differences. No parts of the data, nor intervention materials, have been published elsewhere.

## Results

A total of five themes, across three stages, were identified (see Fig 1). Themes 1 and 2 relate to the first stage: (1) the responsibility-capacity paradox in managing emotion in the classroom, and (2) the differentiated perceived feasibility and acceptability for the classroom. Themes 3 and 4 relate to the second stage: (3) dual benefit perceived for mental health and learning, and (4) perceived feasibility, practice change and context-dependent scalability. Theme 5 relates to the third stage: (5) endorsement of perceived feasibility, practice change, and scalability.

**Fig 1:**
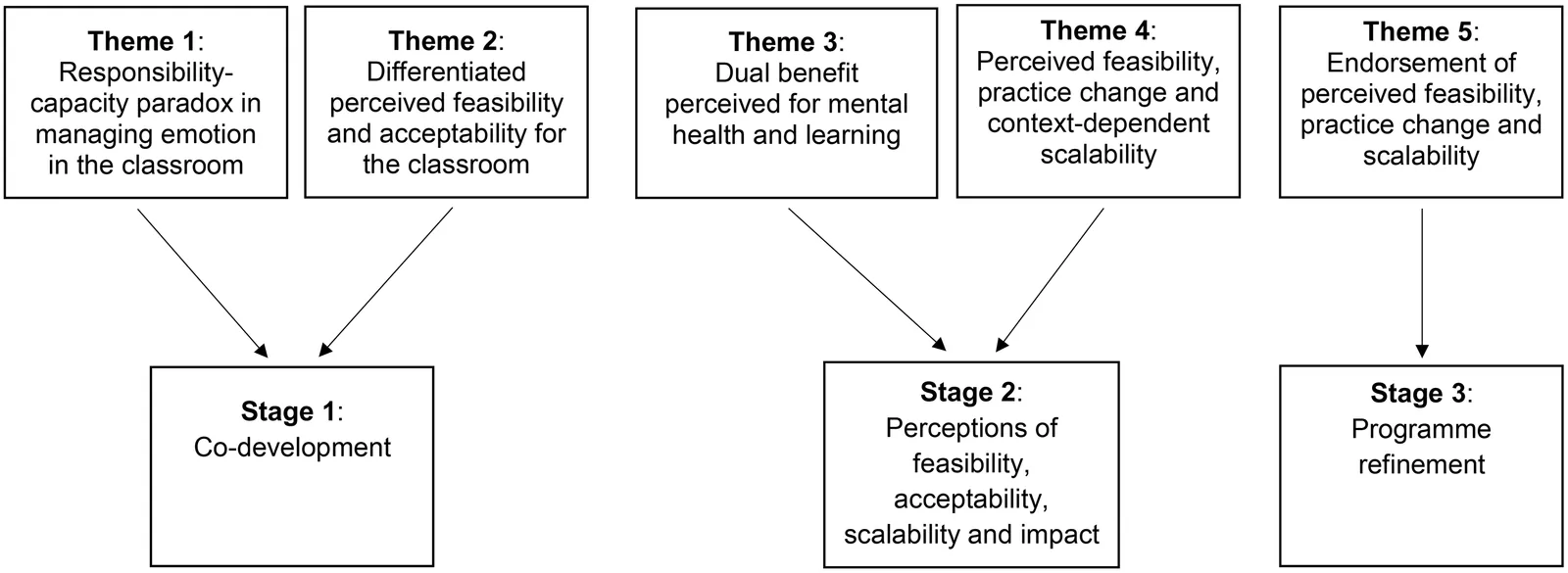
Themes of the intervention development.

### Stage 1: Co-development

#### Terminology, responsibility, priorities, challenges and opportunities.

Teachers used contrasting and dichotomous language to shift between the term ‘mental health’ as negative and ‘wellbeing’ as positive. Teachers expanded their boundaries of professional responsibility as all agreed that part of their role was to support the mental health and wellbeing of pupils, albeit not fully their responsibility alone. Priority areas for teachers focused on managing anxious feelings and supporting emotional understanding and regulation as a core part of their teaching, noting that young people’s emotions tend to escalate quickly and severely. Teachers also highlighted that the mental health needs of pupils are increasing and that more recognition is needed of the sustained impact of mental health symptoms as they present in the classroom. Teachers highlighted the need for dedicated language around mental health and wellbeing as a top priority, and articulated the need for scaffolding in relation to specific resources and training, particularly around identifying and supporting difficulties relating to mental health, supporting emotion regulation, and using appropriate language with young people. Furthermore, teachers identified the need for whole-school approaches and effective leadership that drives forward mental health initiatives for not only pupils, but for staff too.

Numerous challenges in supporting and promoting young people’s mental health and wellbeing in the classroom were noted by teachers, from internal (e.g., high levels of noise, crowded classrooms) to external factors (e.g., the rise in energy drink consumption, to lack of food at home). However, the main structural challenges were difficulties in balancing supporting young people’s mental health with the high workload of pupils and teachers, and systemic gaps in training and mental health literacy (i.e., knowledge about mental health disorders that aids recognition, management, and prevention), and not feeling appropriately equipped to do so. For teachers, opportunities to support and promote mental health and wellbeing in the classroom that focused on the pedagogical integration of ‘hands on’ strategies into routine lessons to address the diverse needs of pupils. The consensus was for a brief, but evidence-based transdiagnostic ‘tool-kit’ approach, particularly driven by high workloads and teacher time constraints.

Overall, a theme emerged from the tension experienced by teachers who actively expanded the boundaries of their professional identity by viewing emotional understanding and support as a core part of their daily job, yet experienced a responsibility-capacity paradox in which they are structurally limited by low levels of mental health literacy, overwhelming workloads, and a lack of training, tools, and structured support to do so effectively. Despite these challenges, teachers identified opportunities to mitigate this paradox that included brief, practical tools embedded into routine lessons that address multiple pupils needs. This tension highlights the need to position emotion regulation as a central pedagogical priority within everyday lessons, which is supported by shared language and leadership that acknowledges and supports the emotional needs of both pupils and teachers. The theme and sub-themes with verbatim examples can be found in Table 5.

**Table 5:** Responsibility-capacity paradox in managing emotion in the classroom (theme 1).

Sub-themes	Verbatim examples
**Dichotomous terminology**	“*it’s often viewed negatively, how do we fix it…*” (P3) “*…wellbeing is like ‘well’, like it sounds positive”; “… in that good mindset*” (P4) “*wellbeing…you know that we’re all in that good mindset…mental health… I think that’s probably a bit where we would probably look to an expert or somebody, like, maybe it’s somebody from CAMHS coming into the school…*” (P5)
**Teacher-identified priority areas**	“*anxiety…that is very commonly presenting*” (P2) “*they [pupils] are quick to say they are really anxious or really depressed*” (P3) “*there are so many that are really struggling, so it’s maybe not coming to school or missing certain classes or having panic attacks during class as well and just feeling overwhelmed*” (P6) “*I think the learners maybe need to understand…it’s OK to feel sad a little bit. It doesn’t mean you’re depressed…*” (P3) “*helping them understand that there’s alternative ways to be thinking about things and dealing with things*” (P3) *“they [pupils] don’t have the emotional literacy to even know what they’re feeling…”* (P4) *“it seems to be jumping, a lot of, not steps, not saying you have steps towards that but, a lot more of them are coming in with self-harming about talking about wanting to harm themselves”* (P5) *“…they [pupils] bring stuff with them and we’re not necessarily equipped in that space.”* (P9) *“… there has to be a true recognition now that this [mental health symptoms] is a real thing affecting every day, every period of a lesson….* (P2)
**Expanding boundaries of professional responsibility**	*“that’s [mental health] a huge part of what we do, but it isn’t solely our responsibility”* (P1) “*it is actually a teacher’s responsibility to really know the young people in their classroom*” (P2)
**Articulating teacher needs for scaffolding**	“*training on the conversations… and the support that you could give…*” (P4) “*strategies we can come up with that will allow them to move, maybe that little bit closer to coming in [to the classroom]”* (P2) “*I think there has to be a true recognition now that this is a real thing affecting every day, every period of a lesson…”* (P2) *“… with really supportive leadership team who know how to drive some of these things, was more likely to have positive outcomes.”* (P2) “… *staff mental health and wellbeing really needs to be like a key part of this as well, so that everyone then can benefit*” (P3)
**Structural challenges in teacher capacity**	“*you can’t focus on any one person for any length of time… it’s very difficult to, have meaningful check-ins*” (P4) “*it’s time because you don’t have the time*” (P8) “…*we have so little time and so much to get through. It’s balancing that with giving them time just to breathe and to not be stressed*” (P1) “*… they bring stuff with them and we’re not necessarily equipped…*” (P9) “…*for the learners or for ourselves as well… there’s a lack of understanding*” (P3) “*[interventions] will say things like they need a brain break. What does that mean? What can I give them?...it would be good to have a kind of overall, you know, guidance on what that actually looks like and what works particularly well for students with particular needs*…” (P4)
**Pedagogical integration opportunity**	“*So from my perspective, we are looking at very hands on strategies… how do I support the overall wellbeing of the kids in front of me in the class*” (P1) “*In an English classroom, you’re fortunate in the texts that we study sometimes lend themselves to discussions where we can talk about wellbeing and things*” (P1) “*I think having a toolkit for those kind of conversations would be really beneficial…*” (P10) “*it begins our relationship with them*” (P7) “*I think it might be useful to have a brief summary of some strategies that really help, I’m not saying for every young person, but for say a majority of young people*” (P2) “ *like a pocketbook…or a line of sentences, I can follow*” (P10)

(P) = Participant number.

#### Development of intervention components with teachers and young people.

Synthesising teacher priorities, challenges and opportunities identified in workshop 1 with existing empirical research literature and advisory group consultation, a suite of broad evidence-based strategies (e.g., movement, flexible thinking, problem solving, balancing positive and negative self-talk, gratitude, mindfulness, grounding, and reflecting on strengths, muscle relaxation) was presented to teachers (in workshop 2) and subsequently to young people (in workshop 3). These were accompanied by brief psychoeducational overviews detailing ER, its relationship to mental health, and the importance of goal-directed behaviours and situationally appropriate ER strategies. Teachers and young people engaged in collaborative discussions to co-develop and adapt a range of strategies into routine teaching activities to support and promote mental health and wellbeing.

**Movement**. Teachers recognised the benefit of movement for pupil mental health and all agreed that they would like to add more movement into their classrooms and within the wider curriculum, despite identifying time constraints as a primary barrier to implementation. Teachers and young people converged on the value of replacing passive note-taking in class to embedding real-life, relatable and creative curriculum-embedded movement tasks (e.g., re-enacting parliament in modern studies class). This shared preference included utilising outdoor spaces for movement (e.g., measuring items in physics rather than copying down information or using surrounding streets for treasure hunts and relay races and for end of topic revision). Furthermore, teachers and young people identified optimal structural windows in double periods for introducing active breaks. However, perceptions of implementation highlighted key areas of divergence. While teachers emphasised competition to drive engagement in movement, young people raised concerns that movement to locate answers could cause confusion and distraction. Moreover, while both teachers and young people highlighted pupil self-consciousness as a barrier to implementing movement, teachers focused on repetition to build confidence and enjoyment, whereas young people emphasised that movement must be embedded as a shared whole class activity to make it more comfortable for them to take part. Young people, however, agreed that they would like more active breaks and physically active learning (i.e., movement aligned with the teaching content) in their classrooms, and that this would boost their wellbeing. Overall, embedding movement was perceived as feasible, acceptable and of recognised benefit when implemented to maximize efficiency through whole-class, creative and curriculum-based design.

**Flexible thinking and problem solving.** Teachers recognised the benefit of incorporating flexible thinking strategies, such as challenging negative or all-or-nothing thinking or taking different perspectives into their classrooms, for both the benefit to teachers and pupils. Teachers and young people converged on the value of utilising realistic scenarios, case studies and characterisation as an effective way to embed flexible thinking and effective problem solving into the classroom, specifically identifying and navigating challenges faced by characters within a text. Teachers emphasised the importance of being, in their words, ‘humorous and silly’ with young people and about the need for strategies to support both up regulation (amplifying emotional experiences) and down regulation (reducing intensity of emotional experiences). Conversely, young people cautioned that personal (e.g., sharing one’s own feelings) or ‘high-stakes’ activities (e.g., related to exams) would be better as individual activities. Overall, embedding flexible thinking and problem solving activities were perceived as feasible, acceptable and of recognised benefit through co-regulation, playfulness, characterisation and low-stakes whole-class design.

**Self-talk.** Teachers recognised the benefit that self-talk, such as affirming and supportive phrasing and altering self-limiting statements could have in their classroom. Teachers and young people converged on the value that self-talk is most effective when authentic and aligned within routine teaching. This shared preference included teacher modelling techniques and using characterisation. However, their perceptions of implementation of self-talk diverged regarding tone and authenticity of language. While teachers emphasised the active promotion of positive self-talk phrasing and optimistic wall statements (e.g., successful famous figures who have had many failures before becoming successful), they stressed a challenge in young people believing it. Conversely, young people actively rejected inauthentic and overly optimistic teacher phrasing and positive wall statements. Young people noted that exaggerated positivity felt unhelpful and instead emphasised that modelling authentic and more balanced language with some realistic positivity, would have a greater positive impact on their mental health and wellbeing. Collectively, their perspectives highlight the importance of strategies that are authentic and aligned with subject content. Overall, self-talk was perceived as feasible, acceptable and of recognised benefit through grounding in modelling realistic authenticity and whole-class design.

**Mindfulness, gratitude, reflecting on strengths and muscle relaxation***.* Teachers recognised the benefit of mindfulness, expressions of gratitude, reflecting on strengths and muscle relaxation activities within the school environment, whilst identifying with time constraints as a barrier to implementation. Both teachers and young people converged on the utility of these strategies in routine teaching, particularly highlighting a needed shift to focusing on their strengths than the current balance towards focusing on weaknesses. However, while there was broad support for the activities, the feasibility and acceptability of these activities were seen as context-dependent on the environment, timing and specific classes/ subjects. Young people rejected public disclosures of reflecting on strengths or expressing gratitude in the classroom. Teachers mirrored the need for contextual boundaries, citing perceived workload concerns regarding routine integration. Instead, they advocated for positioning gratitude and strengths reflections as discrete short summary activities (e.g., plenaries, one-to-one support, personal and social education [PSE] classes or lesson/term transition activities). Similarly, the perceived feasibility of mindfulness and muscle relaxation were deemed sensitive to context and time of day. While teachers suggested mindfulness might be optimal in mornings, with smaller classes and/or with young people with additional support needs, muscle relaxation activities were perceived as more suitable to afternoons. Young people expressed reluctance to tensing muscles (i.e., muscle relaxation techniques) stating they would be more acceptable in classes such as drama or physical education where they align with the focus of the class. Ultimately, mindfulness, expression of gratitude, reflecting on strengths, and muscle relaxation activities feasibility was perceived to be conditional to ad-hoc activities as opposed to routine lessons and acceptability perceived to be conditional on contextually aligned activities and environments.

Overall, teachers and young people expressed a shared consensus that the intervention was perceived as highly feasible and acceptable, that it could be meaningfully embedded into routine classroom activities, provided consideration is given to how different components can be implemented. While movement, problem solving, flexible thinking and self-talk were perceived as flexible and adaptable to embed within routine teaching, there was perceived conditional tension in embedding mindfulness, muscle relaxation, expressing gratitude and reflecting on strengths. Feasibility was perceived to be conditional to ad-hoc activities to protect teacher workload and acceptability was perceived to be conditional on contextually aligned environments, underscoring that not all components hold equal acceptability. Notwithstanding these considerations, young people emphasised the critical importance of teachers understanding and supporting their mental health needs in the classroom. At the same time, teachers showed enthusiasm regarding the potential positive impact of embedding strategies into routine teaching activities, deeming them as “brilliant techniques”, whilst noting that embedding them would require some, but not an excessive amount of planning time. The theme and sub-themes with verbatim examples can be found in Table 6.

**Table 6:** Differentiated perceived feasibility and acceptability for the classroom (theme 2).

Intervention component	Sub-themes	Verbatim examples
**Movement**	**Maximising efficiency through whole-class creative and curriculum-based design**	“*It helps a wee bit if you frame it almost as a competition or a game…*” (T: P1) “*they kind of join in with that [beer pong] because senior pupils can relate more…*[laughs]” (T: P8) “*we re-enact parliament and we debate each other so it’s really active and we enjoy it*… So it’s realistic examples of our subjects.” (YP: P12) “I *know a lot of people, especially in double periods, they get like tired a lot. So it’s just a good time to get outside and have some head space*” (YP: P13) “*they don’t second guess when I’m like, right, stand up and let’s move around because it’s a task and it’s just really interesting that the self-consciousness of it totally disappears”* (T: P3) *“…whereas I think if it’s everyone going out for a little break then, you know, people are more comfortable”* (YP: P3) “*after like a few weeks they just know it and they get into it and they actually enjoy it so much more than just sitting there copying down the information*” (T: P3) “*I think just incorporating it [movement] more and being, like, creative with ideas for each different subject”* (YP: P12) “*I mean just from what I see in my classes a lot of people would use that [whole class moving to think of an answer] as an excuse to go and speak to their friends or to just not be doing work*” (YP: P14) “*I feel there is such a pressure to finish the course, that I don’t feel I have a lot of time I can do things that are a bit more active with them, which I’d really like to incorporate more into just the curriculum in general…”* (T: P10) “…*going out and physically doing things in Science rather than just copying them down…going out and measuring things in physics rather than copying down measurements*” (YP: P11)
**Flexible thinking**	**Framing flexible thinking through co-regulation, playfulness, characterisation and whole-class design**	“*doing it with a bit of silliness and a bit of humour*” (T: P1) “*I understand the problems of trying to talk them down… but I actually have a problem talking mine up”* (T: P4) “…*these would be great for the kids, but even as a teacher, sometimes you’re in that negative cycle of thinking”* (T: P4) “*by making examples of some of your historical characters, like where they’ve kind of failed, and this is how they could’ve…if they thought about it this way*” (T: P4) “*… I feel like scenarios are really like real life and what goes on in schools and that allows people to, maybe in some ways, like, respond like if it was them in that position*” (YP: P13) “*I probably wouldn’t have thought about the worst worst [worst case scenario] until someone else had said that to me and that would make me freak out more*” (YP: P11) “i*t [high-*stakes*] wouldn’t be as a group. It would just be individual” (YP: P15)*
**Problem solving**	**Practising problem solving through characterisation and whole-class design**	“*…a problem a character is experiencing in a text and then get them to identify what the possible obstacles for overcoming that problem for that character are, and what their solutions for the character are*” (T: P1) “*So like in Modern Studies and Politics you could possibly be given a case study about a current politician*…” (YP: P12) “*Like if you’re doing Scottish poetry for English, or on a book, like the author or a poet how you are given a description about them and their personal life and things. If they had a problem we could, like, answer that and ask “oh if you had that problem how would you tackle with it and how do you think that they can get out of the situation*” (YP: P13)
**Self-talk**	**Grounding self-talk in modelling realistic authenticity and whole-class design**	“*we can say everything, but it’s not going to make a difference if they don’t think it*” (T: P3) “*In our department, we’ve got a lot of this [negative to positive statements] on the wall, like the growth mind-set…and really trying to repeat the phrases or maybe on the wall displays, so they’re hearing a lot more, and if you’re talking to them, hearing it a lot more and hopefully over time it will just like embed itself will be a bit better*” (T: P3) “*I’ve got a bit more of a generic famous failures wall [a wall of famous people who had failed before coming famous]... and I think they can kind of link that a bit to themselves. I may refer to it at some point and say, “You’re not a failure, look at all these people who said they were failures, and they’re very well-known now*” (T: P10) … *it’s not just something you can display on a wall. It’s something that the teachers can exude, if they are always positive and neutral about it and hopefully it rubs off on us*” (YP: P12) “*Praising the smallest of things, just to boost them a little bit of boost their morale, because I think going through their day must be really hard and they must be exhausted from the end of the school day*…*just trying to pick up on any glimmer of positives that I can*” (T: P6) “*…model the language, so really consciously making that effort…and hopefully over time it will just like embed itself*” (T: P3) “*Sometimes when people say “you can do it, you can do it” it can come off as fake and it can make you doubt yourself a bit more*” (YP: 16) “*I think when teacher is overly positive it comes across as quite cringe. And that just makes people, you know, just urg! So I think neutral talk or even just like reminders every now and then, telling the class, you can actually do this, just not being totally positive like “your all just gonna get an A” yeah, yeah, just being like more neutral*” (YP: P14) “*…encouraging neutral talk would be better as when they [teachers] are overly positive, then anyone who has a low mood just thinks “please shut up” in the nicest way. Whereas if it’s just neutral and its not overly negative where it could make people feel worse but too positive and pupils won’t listen to you so I think neutral is better than being positive*” (YP: P11)
**Mindfulness**	**Confining consciously performed somatic practices to contextually aligned activities/environments**	“*I have classes that I think would engage really well with this first thing in the morning, but trying to do this after lunchtime would be carnage*” (T: P1) “*smaller classes that’s a more nurturing setting and it would maybe be quite useful in those when they come into the department*” (T: P4) “*be quite good for pupils that have additional support needs*” (T: P5)
**Muscle relaxation**	**Confining consciously performed somatic practices to contextually aligned activities/environments**	“*Probably afternoons, I would probably find that that might be something more beneficial, as the day goes on…actually getting them to maybe relax a bit more in the afternoon might be quite an interesting thing to see if that changes sort of the mood or the morale of the class*” (T: P5) “*I think people might be reluctant to squeeze all our muscles up in front of people. I think the teachers might be like “let’s do it” and in reality no one will because you’d look stupid*” (YP: P11) “*I think it would work in practical lessons like drama... I think in English lessons and things like people wouldn’t want to do it”* (YP: P17)
**Expressing gratitude**	**Confining gratitude practices to contextually aligned and discrete summary activities/ environments**	“*Finding the time to do that [expressing gratitude] is going to be really hard. We have 45 minute periods and by the time you get the kids in and settled, realistically you might only get sort of 35 minutes of really strong teaching time. So the idea of taking time away from that just to look at gratitude, I think I would, being honest, I would be really hard pushed to sell that to senior management as well in terms of being something valuable*” (T: P1) “*maybe as a plenary activity, three things that you are grateful that you learned in this lesson*” (T: P1) “*even if you’d be acknowledging that these are not maybe day-to-day classroom strategies, they are still good things that you could probably pitch or use if the opportunity arises with children”* (T: P4)
**Reflecting on strengths**	**Confining reflecting on strengths to contextually aligned and discrete summary activities/ environments**	“*you want to know what their struggling on and so you automatically just fixate on that and they fixate on that [as opposed to strengths]”* (T: P3) “*Something we could do like every couple of weeks just like a 5 minutes at the end of the lesson and things like that. I think we’d be able to find some time*.” (T: P10) “*Especially end of terms, end of topics and stuff*… *as like a plenary, or doing it as a starter is yeah, super straightforward and super easy to produce*” (T:P3)
**General perceived feasibility and acceptability**		“*they sound quite manageable definitely*” (T: P4) *“it does obviously take a bit of planning to adapt your lessons, but generally speaking you’ve got lessons that you use each year… so it’s just about kind of going back and still using the same lesson but just finding ways to bring this into it as well* (T: P3) “*I’m quite excited to see the potential impact that they can have*” (T: P1) *“I think they’re all brilliant techniques, even the ones that can’t be used, or aren’t as able to be used every day, because as you said, there’s only certain amount of teaching time, but it doesn’t mean they’re not usable or applicable with kids that age.”* (T: P3) “*I think every teacher should be made aware that mental health is really important*” (YP: P13) “*You should be able to approach every single teacher and be comfortable to talk to anyone about anything. It seems like some teachers just have this terrible mindset when it comes to mental health and I think*” (YP: P13)

(T) = Teacher from virtual workshop; (YP) = Young Person; (P) = Participant number.

### Stage 2: Perceptions of feasibility, acceptability, scalability, and impact

Following the co-development workshops, the research team refined a list of easily adaptable activities based on evidence-based strategies. These comprised of problem solving (e.g., pupils identifying problems and resolving obstacles faced by characters in a text); movement (e.g., integrating movement into learning such as representing tectonic plate movements); flexible thinking (e.g., using emotive historical situations to gather evidence, evaluate alternative interpretations, and map best, worst, and most realistic scenarios); self-talk (e.g., analysing characters’ dialogue to identify possibilities for more neutral/realistic self-talk or converting negative self-talk into questions); and combining strategies (e.g., merging self-talk with movement by acting out scenes using a character whilst discussing the dialogue). Based on further discussions with teachers and young people additional activities were also developed. This included activities related to opposite action (i.e., consciously behaving counter to an automatic emotional urge) and communication techniques such as the ‘OARS’ model (i.e., Open-ended questions, Affirmations, Reflective listening, and Summaries). While a communication technique, it also serves as a framework to help discuss and explore emotional challenges and thus build ER capabilities in the classroom. These were then integrated into specific classroom activities by teachers across a range of school subjects, and trialled over a four-week period. Teachers then reported back on any perceived impact.

A strong convergence across strategies regarding their perceived impact, with teachers reporting a dual positive impact on young people’s mental health and wellbeing and learning was reported. A clear pattern emerged where activities were perceived to mitigate dysregulated behaviour and observable distress, and perceived increases in wellbeing, classroom focus, motivation and overall academic productivity, and attainment. Teachers reported that the activities were usable and feasible to incorporate into their routine lessons and frequently noted that the activities appeared to be acceptable to young people through their perceived enjoyment, which facilitated initial engagement and sustained participation. Of note, not all activities were equally attempted by teachers, with movement, flexible thinking, problem solving, and self-talk most commonly embedded into routine teaching activities whereas strengths reflections, gratitude, muscle relaxation, opposite action or OARS were attempted once and as one-to-one, or ad-hoc activity (e.g., recording strengths after a topic) or used with specific cohorts (e.g., young carers).

Teachers affirmed feasibility and scalability of the intervention, detailing noticeable shifts in their practice. This practice change was characterised by a deliberate and conscious effort to embed strategies more consistently into their routine lessons, alongside heightened self-awareness of how to best utilise classroom time. Specifically, teachers reported a more conscious effort regarding the language they use, an adoption of more realistic thinking approaches and explicit plans to incorporate physically active learning into their classrooms. In terms of scalability, teachers universally viewed the activities as scalable, working well across year groups and subjects, and providing a strong resource bank of tools to use in the classroom. However, some strategies such as flexible thinking and realistic self-talk were perceived as particularly well suited to subjects with reflective or narrative content (e.g., English, History) and some better suited to individual working due to sensitivities around sharing information (e.g., gratitude, strengths reflections). Teachers stated that overall the activities were accessible, usable, feasible, and scalable, with two themes emerging: 1) dual benefit perceived for mental health and learning; and 2) perceived feasibility, practice change and context-dependent scalability. Themes with verbatim examples on perceived impact can be found in Table 7.

**Table 7:** Dual benefit perceived for mental health and learning (theme 3) and 4 perceived feasibility, practice change and context-dependent scalability (theme 4).

Themes	Verbatim examples
**Theme 3: Dual benefit perceived for mental health and learning**	“*more energetic, more engaged and focused, on their learning*…*they seemed more confident in what they were doing…they don’t seem to be as stressed coming into my class… they get into it and they actually enjoy it so much more than just sitting there copying down the information*” (P3: movement activity) “*… they’ve actually been getting up and doing them, so it’s worked really well for their attainment as well as their wellbeing… I was a bit worried… that they wouldn’t settle back to the tasks afterwards, but no, they were really happy*” (P6: movement activity) “*them actively saying to me, ‘I actually feel a bit better now’….it does just make a wee difference and last period of the day, we actually get some work done.*” (P10: movement activity) “*I felt it gave them a bit of confidence…They seemed to react more positively to situations, I felt, and they didn’t seem to have all the anxiousness that they would normally have.*” (P2: flexible thinking activity) “*Certainly as an approach, I think that they quite liked that they had something almost in front of them that they could look at and say, “right, OK, the worst that’s going to happen is this, which would maybe be really bad, but actually, how likely is that to happen?”* (P1: flexible thinking activity) *“they don’t seem as anxious for the assessment they’ve got…[pupils] feeling a bit more confident, that they could overcome those challenges. So it felt a lot more like things were not so concerning to them.”* (P3: self-talk activity) *“…isn’t so anxious when he comes into the classroom. He definitely comes in much happier.”* (P6: self-talk activity) *“it’s having a more positive impact on how they might carry forward with it [tasks]”* (P4: self-talk activity) “*They really enjoyed getting to do each of the little categories and then kind of sharing it with the class and things.*” (P10: mindfulness activity) “*It felt like a really calm, productive environment…they settled much more easily during the period…*” (P1: muscle relaxation activity) “*…for the kids who weren’t always getting heard…I think that for them was just boosting their confidence… just seeing them actually quite happy, and when they come back next week again, more willing to work with you.”* (P10: expressing gratitude activity) *“So what we did was we talked about the booklets [school task], and they record their strengths from their assessments, but also next steps….so what they ended up with is a little checklist, which felt a lot more like a set of steps that they could go away and follow… I think it left them feeling a bit more confident, that they could overcome those challenges.”* (P1: strengths activity) *“…one of those young people came to me and said, that after the conversation…they were taking that positive step themselves to identify that was the next step for them to try and improve their wellbeing…they look much, much happier and they don’t look as teary, they’re more open. I would say their wellbeing has improved, they really are much happier, and it’s really nice to see that change in them”* (P2: OARS activity) *“It actually completely diffuses the behaviour and the frustration and the stress and we don’t get the big meltdown.”* (P7: opposite action activity)
**Theme 4: Perceived feasibility, practice change, and context-dependent scalability**	*“I feel they are usable as they can be done whilst teaching in the lesson without having to necessarily remove someone from class to do so.”* (P2) *“I feel that there are a number of strategies that can easily be incorporated into the classroom setting without adding to workload pressure or teaching time restraints.”* (P6) *“Some more than others definitely usable without any additional work/prep…I think perhaps the strategies where thoughts and feelings are perhaps easier to use in subjects with similar content (e.g., English and History) with character feelings used as examples to then discuss compared with geography where it wouldn’t fit as readily.”* (P3) *“Very scalable and could be used across the curriculum and year groups.”* (P10) *“Very adaptable.”* (P4) *“some S1s may feel too embarrassed/ shy to share that sort of information with their peers and teachers.”* (P5) *“I do believe that the strategies provide a strong resource bank that teachers can adapt to meet the needs of their own classes and settings, which is as much as any single resource can do.”* (P1) *“I have been working on incorporating a range of strategies into my lessons and will continue to do so in the future.”* (P3) *“It’s really made me think about what I do, and how I do it, and just how spending 5 minutes doing something a little bit different can make a big impact, so I’ll definitely be trying to incorporate a lot more in.”* (P6) *“I have tried making lessons more active for our young people. It has also made me think more about the way in which I communicate with my learners such as the language I use.”* (P8) *“I have become more aware of the positive impact of the strategies both for classwork and for support tutorials.”* (P7)

(P) = Participant number.

#### Impact on teacher mental wellbeing, mental health literacy and perceived stress.

To examine any pre-post changes of taking part in the four development and evaluation workshops, teachers were assessed on pre-and-post mental wellbeing, perceived stress and mental health literacy measures. Shapiro-Wilk assessment of the distribution of paired difference scores, which is the relevant normality assumption for the paired *t*-test, confirmed normal distribution for mental health literacy and stress (*p* > .05), however mental wellbeing significantly deviated from normality (*p* = .01). Consequently, a non-parametric Wilcoxon signed-rank test was used to evaluate mental wellbeing, while remaining outcomes were analysed using exploratory paired-samples *t*-*t*est. To control for the inflation of Type 1 errors across multiple comparisons, a Bonferroni correction was applied across the three exploratory inferential comparisons, adjusting the significance threshold α = 0.0167 (0.05). A Wilcoxon signed-rank test indicated that there was no significant differences from pre (*Mdn* = 22.50, *IQR* = 4.15) to post (*Mdn* = 23.50, *IQR* = 5.5) workshop on teachers’ mental wellbeing scores *Z* = −.718, *p = .472* (two-tailed), *r* = −.16, with a Hodges-Lehmann median difference of 0.50, 95% CI [−1.00, 4.00]. Conversely, parametric paired samples *t*-*t*ests showed no significant difference in mental health literacy scores from pre (*M* = 140.80, *SD* = 7.27) to post workshops (*M* = 141.60, *SD* = 10.09), with a mean difference of −0.80 (*SD*_diff_ = 6.78, *SE* = 2.14), *t*(9) = −.373, *p = .*718 (*t*wo-tailed), 95% CI [−5.65, 4.05]. The standardised effect was small, *d*_*z*_ = 0.118, 95% CI [−0.737, 0.507]. There was no significant difference in teacher stress scores from pre (*M* = 19.60, *SD* = 6.87) to post workshops (*M* = 16.50, *SD* = 6.02), with a mean difference of 3.10 (*SD*_diff_ = 6.23, *SE* = 1.97), *t*(9) = 1.574, *p = .*150 (*t*wo-tailed), 95% CI [−1.35, 7.55]. The standardised effect was moderate, *d*_z_ = .498, 95% CI [−0.174, 1.146]. Despite the observed mean reduction of 3.10 points, the difference was not statistically significant and the wide confidence internal indicates substantial uncertainty. Taken together, these findings suggest that no statistically detectable pre-post differences were observed from taking part in the workshops and the findings remain exploratory.

### Stage 3: Programme refinement

Following the initial four development, feasibility and evaluation workshops with teachers and one development and feasibility workshop with young people, a sixth workshop with a new set of teachers who were not involved in the initial development and evaluation was held. Feedback was sought on the activities’ perceived feasibility, acceptability and impact of embedding strategies in routine teaching activities, as well as to co-develop any new adaptations, or new additions to, the activities specific for application in routine teaching.

The strategies were deemed as acceptable, usable and accessible for routine teaching. Teachers described intentions to change aspects of their practice, including plans to embed activities into their practice, adopt a heightened awareness of classroom language, and increased modelling of realistic positive self-talk language in class. In terms of scalability, while teachers confirmed universal curriculum-wide potential across year groups such as incorporating realistic thinking, physically active learning and flexible thinking approaches across subjects, they emphasised that successful scaling relies heavily on the structural design of the intervention materials. To maximize practical usability, they advised that a final classroom resource must be brief, specific and easy to navigate tool-kit, utilising materials small sections and where possible supported by bullet points that can be operationalised within the fast pace of the school day. Similar to the initial development stage, some considerations were noted in relation to the sensitivities around young people sharing personal information and some teachers suggested some strategies were particularly well suited to subjects and activities with reflective or narrative content (e.g., English, Drama, PSE). Overall, a theme emerged of perceived endorsement of feasibility, intended practice change, and scalability. The themes with verbatim examples on can be found in Table 8.

**Table 8:** Endorsement of perceived feasibility, intended practice change, and scalability (theme 5).

Theme	Verbatim examples
Endorsement of perceived feasibility, practice change, and scalability	*“A lot of ideas seemed to be applicable to a wide range of subjects but, as a maths teacher, many just don’t apply in the maths class so was great to see specific examples relating to maths too.”* (P26) *“Very usable, before I felt like you needed to talk about mental health specifically to use in lessons but actually you can teach strategies through other concepts that help young people to for example problem solve.”* (P25) *“I feel like there were lots of easily accessible suggestions. I’d be willing to try many of the things we discussed and will definitely implement some of them.”* (P22) *“I am going to watch my language and how I interact with the classes. I am going to avoid using “I should/shouldn’t” statements and watch how I engage with a pupil when they are negative. I already use “What evidence do you have to support this?” However, I am going to ensure that I am not speaking negatively and absolutely adopting “think realistic,” regarding all situations.”* (P23) *“I plan to use the best case, worst case, most realistic scenarios when working with young people to help them think more rationally.” (P35)* *“All the strategies could be used for all year groups, no problem with that.”* (P30) *“I can see how in English/drama there may be more opportunity to use these strategies regarding a text/role play.”* (P23) *“…might put young people off sharing in groups”* (P22) *“I think they are very usable especially in science and biology”* (P33)

(P) = Participant number.

## Discussion

The current paper set out to report the findings from the development phase of a co-developed transdiagnostic mental health intervention, developed for use in routine teaching in secondary school classrooms: The CAMH Class intervention. This followed the updated MRC framework for developing complex interventions [60], and the INDEX study guidelines [61]. Using a partnership and co-production approach with teachers, young people, researchers and clinicians, the current project brought together multiple professions and disciplines, across a range of media and methods. This allowed for a range of viewpoints, the identification of challenges and opportunities, as well as perceived impact, practice change and scalability for a variety of classroom settings. Specifically, for school-based interventions, where complexity exists and understanding of the complex psychosocial context imperative, this process provided rigorous, in-depth understanding of the psychosocial context, perceived feasibility, acceptability, and applicability from the people who will use and receive the intervention. This development work identified teacher mental health priority areas in the classroom and linked these to mental health strategies, extending past work in an important way by embedding them directly into routine teaching activities.

During the initial co-development stage, a responsibility-capacity paradox was identified (theme 1). Teachers viewed the supporting and promoting the mental health needs of their pupils as a core part of their role, yet faced systemic constraints of low levels of mental health literacy, overwhelming workloads, and a lack of training, tools and support to do so effectively. Despite these challenges, teachers identified opportunities specifically by advocating for brief, pragmatic universal tools that can be embedded into routine lessons to accommodate diverse pupil needs, and supplemented by a shared language and leadership that actively supports the emotional needs of teachers and pupils. Teachers and young people expressed a shared perceived consensus that the intervention was feasible and acceptable, provided consideration is given to how different components can be implemented (theme 2). This drew distinctions between perceptions of whole-class and every day embedded activities (e.g., movement, flexible thinking, problem solving, self-talk strategies) and conditional or ad-hoc activities (e.g., mindfulness, expressions of gratitude, reflecting on strengths and muscle relaxation strategies). Based on young people’s and teachers’ feedback, conditional and ad-hoc activities were adapted to ensure alignment with the focus of the class (muscle relaxation), and where necessary as individual and non-shared exercises (e.g., strengths, gratitude). Next-phase testing will evaluate whether these activities can be better incorporated into whole-class delivery through creative pedagogical tools, such as the use of relatable characters, guided stories, or illustrative metaphors to increase acceptability or ultimately these will be removed from the intervention.

Crucially, when teachers embedded activities into their practice, there was a dual benefit perceived for mental health and learning (theme 3). This included perceived reduced dysregulated behaviours, perceived increases in wellbeing, classroom focus, motivation and overall academic productivity and attainment, as well as teacher-reported pupil acceptability through the perceived enjoyment of activities. Teachers affirmed perceived feasibility, practice change and context-scalability of the intervention (theme 4), noting activities scaled well across year groups and subjects and were particularly well suited to subjects with reflective or narrative content (e.g., English, History) with some better suited to individual working due to sensitivities around sharing information (e.g., gratitude, strengths reflections). A final development refinement loop with a new group of teachers confirmed perceived feasibility, intended practice change and scalability (theme 5). They noted that strategies could be scalable across the curriculum and year groups, with some noting similar considerations around individual activities and strategies and others being particularly well suited to subjects and activities with reflective or narrative content.

Engaging with teachers and young people in the development of the intervention allowed an in-depth understanding of how the activities were experienced and whether they would be enjoyable and acceptable to young people. Adopting this approach allowed for a balanced method of co-design by combining techniques together with teacher and young people experts to ensure perceived feasibility and acceptability in the classroom, which research has suggested may be more effective than purely researcher-developed top-down programmes [89]. A recent review reported that teachers observing a positive impact on pupils’ engagement and wellbeing is a key facilitator in the sustainability of school-based interventions [90]. Perceived benefits of interventions by school staff have also been noted as a facilitator to sustainability [51], although further research with a longitudinal design is needed to develop a thorough understanding of sustainability over time. There were also no adverse/unintended consequences from taking part in the workshops, which will continue to be a consideration in next phase development given the possible adverse/unintended effects noted in school-based programmes [91].

The resultant intervention is a universal programme, ready for refinement and further testing, to be delivered by secondary school classroom teachers as part of routine lesson activities. The intervention could be applied across multiple classroom subjects and year groups, and will be ready for subsequent evaluation of its effects on young people’s skills to support and promote their mental health and wellbeing. This approach is unique compared to existing universal school-based interventions, Social and Emotional Learning (SEL) programmes, and modular transdiagnostic psychotherapy in its mode of delivery and positioning. The current intervention operates by embedding directly into routine lesson plans across multiple subjects (e.g., English, Mathematics, Physical Education, Geography) and is delivered by all classroom teachers, in contrast to stand-alone fixed, discrete programmes or manualised curriculums. Furthermore, it fundamentally differs from modular transdiagnostic psychotherapies in its target audience. Rather than functioning as a clinical treatment targeting clinical symptoms or pupils directly through specialised deliveries, the current intervention targets teachers understanding and practice, and integrates within routine with classroom pedagogy. Positioning the intervention within a whole-school framework ensures that individual psychological skill-building is complemented by environmental and structural supports within the school ecosystem.

By shifting the focus towards systemic routine-based teaching, the intervention translates core psychological mechanisms into a practical framework compatible with the day-to-day classroom environment. It is considered a brief intervention when compared to other school-based programmes, as some of the activities could be completed in a few minutes, and may be more welcomed by school staff who have continuously noted time constraints as a barrier to implementing school-based mental health interventions [51,92]. The optimum frequency of delivery of the CAMH Class intervention, however, will be examined in next stage testing in order to provide concrete recommendations. It also goes some way to addressing practical and time resource difficulties, the incompatibility and sustainability beyond the conclusion of traditional interventions, and stigma around taking part in mental health related interventions that has been noted elsewhere [39,50,51,53]. The CAMH Class intervention has potential to be used alongside or in combination with other interventions, where more intensive or indicated treatments are warranted.

### Strengths and limitations

This study provides a rigorous and in-depth understanding of the psychosocial context and acceptability from the people who will use and receive the intervention. Whilst other interventions have been developed by researchers, this is the first to our knowledge which has applied a rigorous co-production approach engaging young people and teachers, and aligning the activities with contemporary understanding from research evidence into routine teaching. While the sample included voices from elected MSYPs who represent the voice of young people across Scotland and also a number of teachers from a broad range of subjects and local authority areas in Scotland, it is important to acknowledge that the small sample included an over-representation of females and an under-representation of young people from the most deprived areas in Scotland. It is also important to acknowledge potential volunteer bias and possible social desirability effects which may impact on our conclusions. This may impact the generalisability of the findings and will be a consideration for next stage development. Future development will also continue to embed co-design models to enhance shared decision-making and ensure broader representativeness across the intervention lifecycle. In addition, while beyond the aims of the current study, a further limitation is that no objective measures directly examining pupil mental health or impact on education were administered, and measures focused solely on qualitative perceptions from teachers and young people. Quantitative mental health and education outcome data will be included in next phase testing.

Ultimately, the effectiveness and sustainability of any intervention is dependent on robust and thorough developmental work [60]. Taking a systems-based approach, it was recognised that the development, implementation and sustainability of the intervention, is likely dependent on a wide range of factors which include teachers’ and young people’s beliefs, motivations and capabilities, the wider school structures and environment and the interactions between them. The idea of supporting and promoting mental health in schools is complex and part of a larger, interconnected whole, and this development work helps to understand the networks of interactions to inform the development and evaluation of the intervention and allow us to refine the programme theory for next stage testing. Viewing school-based interventions, particularly those embedded into routine teaching, through a systems lens has the potential to identify more effective ways to improve the mental health and wellbeing of children and young people. Delivering an intervention into a complex system, such as a classroom environment within a dynamic and multi-faceted school system, is challenging with many possible barriers. While the current study highlighted valuable insight into some of these barriers, as well as opportunities, further work is needed to provide a greater understanding of the complexity inherent in trialling the intervention beyond the development group. Following the MRC framework [60], further trials of the CAMH intervention will be undertaken with teachers not involved in the initial development phase, with outcomes including diagnostic and symptom level data of mental health difficulties. As the current study focused on the development phase, formal implementation science parameters such as fidelity, dose, adaptation tracking and sustainability planning were not quantifiably measured. However, these parameters have been integrated into the protocol for our next-phase testing of the intervention, aligning with the RE-AIM/PRISM framework.

## Conclusion

The findings reported highlight vital considerations for designing and implementing school-based interventions, beyond the current intervention, and have the potential to bridge the gap in intervention translation to the classroom. Specifically, the current study allowed mapping of the development of the intervention through co-design, facilitators and barriers to implementation from user and recipients, and the identification of a number of areas needed for refinement before the development of the finalised intervention protocol. The next steps are to refine the intervention, including the programme theory, and develop an intervention protocol ready for testing in schools which will include evaluation of the impact on ER and pupil mental health. Given that effect sizes for school-based preventative interventions tend to dissipate over time [42] the next phase plan is to determine how often and which activities should be implemented, across which ages, stages and classrooms, and whether refresher researcher-led training sessions are needed to maintain the intervention effects. Continued collaborative working and decision-making with teachers and young people within school systems will be key in facilitating the successful delivery of the CAMH Class intervention. This will lead to an intervention, ready for implementation, which has been co-produced, with systems-level thinking, with the aim to improve the mental health landscape in schools by enhancing pupil ER and mental health.

## Supporting information

S1 Table Semi-structured focus group interview questions. (PDF)

S2 Table Coding tree. (PDF)
