# Supplementary material for: Co-developing the Child and Adolescent Mentally Healthy Classroom (CAMH Class) intervention for use in routine teaching in secondary schools
Source: PLoS One. 2026 Oct 12;21(10):e0359563. doi: 10.1371/journal.pone.0359563 (PMC13657300; doi:10.1371/journal.pone.0359563)
Supplement: Supplementary file 1 [file pone.0359563.s001.pdf]

S1 Table. Semi-structured focus group interview questions

Stage 1: Co-development	Questions
**Workshop 1 (teachers)**	1. What is your understanding of the term "mental health and wellbeing"?<br>2. What areas of mental health do you feel are causing the most difficulty for pupils at the moment and how do they present in class?<br>3. To what extent do you think it is a teacher's responsibility to support and promote pupil mental health and wellbeing in the classroom?<br>4. To what extent do you feel equipped, and confident, to support and promote pupil mental health and wellbeing in the classroom?<br>5. What do you think teachers need to help them feel more equipped and confident to support and promote young people's mental health?<br>6. What would you say are some of the challenges in supporting and promoting pupil mental health and wellbeing in the classroom?<br>7. What would you say are some of the potential opportunities in the classroom to support and promote pupil mental health and wellbeing?<br>8. Is there anything you would like to tell us that you have not yet had the chance to?
**Workshop 2 (teachers)** [Following responses in workshop 1, the research literature and consultation with the advisory group, a range of broad evidence-based strategies, accompanied with emotion related psychoeducation, was presented to teachers: e.g. movement, flexible	1. Would you want to incorporate [strategy] into your routine teaching?<br>2. How might you incorporate [strategy] into routine teaching activities?<br>3. How possible/practical do you think it would be to incorporate [strategy] into routine teaching activities?<br>4. Do you think teachers/pupils would like the activity(s)?<br>5. Do you think teachers/pupils would engage in the activity(s)?<br>6. How might you adapt these to different year groups, different subjects, and different times of the day?<br>7. Is there anything you would like to tell us that you have not yet had the chance to?

thinking, problem solving, balancing positive and negative self-talk, gratitude, mindfulness, grounding and reflecting on strengths, muscle relaxation]	
**Workshop 3 (young people)**<br>[Following workshop 1 and 2, a list of classroom activities was devised and shared with an explanation the strategy it is based upon and accompanied with emotion related psychoeducation]	1. Would you, or do you think any other pupils would, like [activity] in the classroom?<br>2. Would you, or do you think any other pupils would, participate/do [activity] in the classroom?<br>3. How possible/practical do you think it would be to do [activity] in the classroom?<br>4. Do you, or think any other pupils would, want to change [activity] in any way?<br>5. Do you have, or think any other pupils, would have any better or other suggestions to use [strategy] in the classroom?<br>6. Is there anything you, or think any other pupils, would want their teachers to do in the classroom to support and promote pupil mental health and wellbeing?<br>7. Is there anything you would like to tell us that you have not yet had the chance to?
**Stage 2: Perceptions of feasibility, acceptability, scalability and impact**	
**Workshop 4 (teachers)**<br>[Following workshop 3, a list of current, new and/or adapted classroom activities was shared]	1. Would you want to incorporate [activity] into your routine teaching?<br>2. How might you incorporate [activity] into routine teaching activities?<br>3. How possible/practical do you think it would be to incorporate [activity] into routine teaching activities?<br>4. Do you think teachers/pupils would like the activity(s)?<br>5. Do you think teachers/pupils would engage in the activity(s)?

	6. How might you adapt these to different year groups, different subjects, and different times of the day?<br>7. Is there anything you would like to tell us that you have not yet had the chance to?
**Workshop 5 (teachers)** [Following agreement of activities in workshop 4, teachers integrated activities into their lessons over a four-week period and report back in workshop 5]	1. What activities did you choose to embed into your lesson(s)?<br>2. How did you embed the activity into your lesson(s)?<br>3. How possible/practical was it to embed into your lesson(s)?<br>4. What age group(s)?<br>5. What subject(s)?<br>6. What impact, if any, did you notice?<br>7. Would you suggest any changes to the activities?<br>8. Is there anything you would like to tell us that you have not yet had the chance to?
**Stage 3: Programme refinement**	
**Workshop 6 (new teacher group)** [Following workshop 5, a refined list of activities based was shared with a new group of teachers not involved in the initial development]	1. Would you want to incorporate [activity] into your routine teaching?<br>2. How might you incorporate [activity] into routine teaching activities?<br>3. How possible/practical do you think it would be to incorporate [activity] into routine teaching activities?<br>4. Do you think teachers/pupils would like the activity(s)?<br>5. Do you think teachers/pupils would engage in the activity(s)?<br>6. How might you adapt these to different year groups, different subjects, and different times of the day?<br>7. Would you want to change [activity] in any way?<br>8. Do you have any better or other suggestions to use [strategy] in the classroom?
