# Supplementary material for: Co-developing the Child and Adolescent Mentally Healthy Classroom (CAMH Class) intervention for use in routine teaching in secondary schools
Source: PLoS One. 2026 Oct 12;21(10):e0359563. doi: 10.1371/journal.pone.0359563 (PMC13657300; doi:10.1371/journal.pone.0359563)
Supplement: Supplementary file 2 [file pone.0359563.s002.pdf]

S2 Table: Coding tree

Theme and descriptor	Sub-themes	Codes
**Theme 1:** Responsibility-capacity paradox in managing emotion in the classroom<br><br>**Descriptor:** Captures the structural and professional tension experienced by teachers who view emotional understanding, support and regulation as a core part of their professional identity, yet are constrained by low mental health literacy, overwhelming workloads and a lack of training, tailored tools and managerial support. This paradox is mediated by an explicit need for brief, practical tools	Dichotomous terminology	• 'Wellbeing' as a positive / accessible term<br>• 'Mental health' as negative / inaccessible term
	Reframing the teacher-role: priorities	• Managing chronic emotional difficulties in daily lessons<br>• Supporting emotional understanding / regulation as core part of teaching<br>• Essential nature of mental health literacy<br>• Recognition of sustained classroom impact of mental health / distress
	Expanding boundaries of professional responsibility	• Perception of full responsibility for pupil mental health<br>• Perception of partially responsibility for pupil mental health
	Articulating needs for scaffolding	• Need for specified resources / training to support in mental health / wellbeing / literacy<br>• Need for staff mental health and wellbeing support<br>• Need for whole-school effective leadership
	Structural challenges in teacher capacity	• Balancing workload / tension<br>• Systemic gaps in mental health support / promotion training<br>• Systematic gaps in pupil / teacher mental health literacy
	Pedagogical integration opportunity	• Transdiagnostic strategies addressing diverse needs<br>• Practical / 'hands-on' / 'tool-kit' / activities<br>• Embed into routine lessons<br>• Simple language / brief instruction

embedded into routine lessons that address a diverse set of pupil needs.		
**Theme 2:** Differentiated perceived feasibility and acceptability for the classroom<br><br>**Descriptor**: Captures the intervention as feasible and acceptable, with consideration needed on how different components can be implemented into routine teaching. Movement, problem solving, flexible thinking and self-talk are perceived as adaptable to routine teaching, yet mindfulness, muscle relaxation, expressing gratitude and reflecting on strengths are	Movement: Maximising efficiency through whole class creative and curriculum-based design	• Desire / benefit for movement<br>• Time / workload tension<br>• Utilise breaks / double periods<br>• Ground movement in real-life / practical / creative / relatable actions<br>• Outdoor activity opportunity<br>• Whole-class execution for time / workload constraints / decrease self-consciousness
	Flexible thinking: Framing flexible thinking through co-regulation, playfulness, characterisation and whole-class design	• Desire / benefit for flexible thinking<br>• Up / down regulation<br>• Value of co-regulation / playfulness / humour<br>• Personal / high stakes individual<br>• Use realistic scenarios / fictional characters / case studies to model flexibility<br>• Whole-class execution for time / workload constraints / decrease self-consciousness
	Problem solving: Practising problem solving through characterisation and whole-class design	• Desire / perceived benefit for problem solving<br>• Use realistic scenarios / fictional characters / case studies to model problem solving<br>• Whole-class execution for time / workload constraints / decrease self-consciousness
	Self-talk: Grounding self-talk in modelling realistic authenticity and whole-class design	• Desire / perceived benefit for authentic / realistic talk<br>• Rejection / ineffectiveness of overly positive wall / teacher statements<br>• Teacher modelling focused on realistic positivity / neutral

perceived to be conditional / context-dependent to protect workload and self-consciousness of young people.		• Use realistic scenarios / fictional characters / case studies to model authentic and realistic self-talk<br>• Whole-class execution for time / workload constraints / decrease self-consciousness
	Mindfulness: Confining consciously performed somatic practices to contextually aligned activities / environments	• Desire / perceived benefit for mindfulness<br>• Time / workload tension<br>• Context-dependent: mornings / small classes / additional support settings<br>• Rejection of sharing responses with full class
	Expressing gratitude: Confining gratitude practices to contextually aligned and discrete summary activities / environments	• Desire / perceived benefit for gratitude<br>• Time / workload tension<br>• Context-dependent: 1-2-1 / personal and social education lessons / plenary / ad-hoc activity<br>• Rejection of sharing responses with full class
	Reflecting on strengths: Confining strengths practices to discrete summary activities / environments	• Desire / perceived benefit for strengths<br>• Time / workload tension<br>• Context-dependent: plenary / ad-hoc activity / end of term / end of topics<br>• Rejection of sharing responses with full class
	Muscle relaxation: Confining consciously performed somatic practices to contextually aligned activities/environments	• Desire / perceived benefit for muscle relaxation<br>• Time / workload tension<br>• Context-dependent: practical classes / drama / physical education / afternoons<br>• Rejection in doing front of peers
**Theme 3:** Dual benefit perceived for mental health and learning<br><br>**Descriptor:** Captures that the intervention is perceived as feasible and acceptable, with perceived dual impact on mental health and learning. It demonstrates that, where tested, activities mitigated		• Pupil acceptability / visible enjoyment<br>• Selected components / use of strategies<br>• Observable mitigation of dysregulated behaviours /distress

dysregulated behaviours and alleviated distress, which fostered a calmer classroom environment conducive to increased focus, motivation overall productivity, visible enjoyment and participation.	• Observable impact on learning / motivation for / attainment<br>• Observable impact on environment / classroom climate
**Theme 4:** perceived feasibility, practice change, and context-dependent scalability<br><br>**Description:** Captures perceived validation of perceived feasibility, scalability and practice change. It captures shifts in pedagogical practice, manifested as a deliberate effort to embed strategies into routine lessons, along with a heightened self-awareness regarding language. While universally valued as a resource bank, scalability is context-dependent, with specific activities requiring intentional tailoring based on subject matter or student year group.	• Feasibility / acceptability validation<br>• Practice change / intentional shifts in pedagogy / self-awareness<br>• Lack / limited workload tension<br>• Universal validation of intervention<br>• Context-dependent scalability / natural alignment with reflective / narrative subjects / decrease self-consciousness
**Theme 5:** Endorsement of feasibility, practice change and scalability<br><br>**Descriptor:** captures external validation from a new teacher group not involved in the initial development. The cohort endorsed the strategies as feasible, acceptable and accessible for routine teaching, promoting immediate intentions to alter and model language. Universal scaling across subjects was confirmed, provided that resources remain brief, structured, easily accessible and minimise pupil disruptions.	• Independent universal verification of feasibility / acceptability<br>• Intentions to alter pedagogy / self-awareness<br>• Endorsement of universal curriculum-based scaling across subjects<br>• Natural alignment with reflective / narrative subjects<br>• Decrease self-consciousness<br>• Brief / accessible resources
